# Case Report: Life-threatening pancytopenia with tislelizumab followed by cerebral infarction in a patient with lung adenocarcinoma

**DOI:** 10.3389/fimmu.2023.1148425

**Published:** 2023-07-25

**Authors:** Hang-Yu Gu, Jing-Wen Zhao, Yin-Shuang Wang, Zhuo-Nan Meng, Xiu-Ming Zhu, Fu-Wei Wang, Ai-Hong Zheng, Guo-Qing Wu

**Affiliations:** ^1^ The Second Clinical Medical College, Zhejiang Chinese Medical University, Hangzhou, Zhejiang, China; ^2^ Cancer Center, Department of Medical Oncology, Zhejiang Provincial People’s Hospital (Affiliated People’s Hospital, Hangzhou Medical College), Hangzhou, Zhejiang, China

**Keywords:** tislelizumab, pancytopenia, hematological irAEs, cerebral infarction, lung adenocarcinoma

## Abstract

Immune checkpoint inhibitors (ICIs) are an integral antitumor therapy for many malignancies. Most patients show very good tolerability to ICIs; however, serious immune-related adverse events (irAEs) with ICIs have been well documented and prevent some patients from continuing ICIs or even become the direct cause of patient death. Cytopenia is a rare irAE but can be life-threatening. Here, we present the case of a 66-year-old male patient with metastatic lung adenocarcinoma who received two doses of chemotherapy + PD-1 antibody tislelizumab and developed pancytopenia after each dose. Although the first episode of pancytopenia resolved with a treatment regimen of granulocyte colony-stimulating factor (G-CSF), thrombopoietin (TPO), and red blood cell and platelet transfusion, the second episode showed extreme resistance to these treatments and improved only after the administration of steroids. His second pancytopenia episode resolved after a long course of treatment with methylprednisolone, G-CSF, TPO, hetrombopag and multiple red blood cell and platelet transfusions. However, he suffered a cerebral infarction when his platelet count was in the normal range and gradually recovered 1 week later. This case highlights the importance of the early recognition and management of hematological irAEs.

## Introduction

Hematological immune-related adverse events (Hema-irAEs) with ICIs are very rare irAEs that manifest as thrombocytopenia, anemia, neutropenia, hypereosinophilia, bi-cytopenia, or even pancytopenia (1, 2). Recently, a French report (3) from the review of cases of ICI-related grade ≥2 cytopenia within the French pharmacovigilance database showed that 68 patients experienced 75 episodes of ICI-related cytopenia which consisted of thrombocytopenia (50.7%), autoimmune hemolytic anemia (25.3%), neutropenia (13.3%), pure red cell aplasia (8%), and aplastic anemia (2.7%). Nearly half of the cytopenia cases were grade ≥4, and 4.4% of patients died from cytopenia-related complications. The incidence for ICI-induced grade 4 neutropenia was reported to be 0.14% in a German melanoma center (4). The median onset and duration of ICI-related neutropenia was 10.5 weeks after the first ICI administration and 13 days, respectively (5). The treatment regimen for ICI-related neutropenia mainly included granulocyte colony-stimulating factor (G-CSF) and intravenous corticosteroids (4). However, patients with ICI-related thrombocytopenia were reported to be associated with worse overall survival compared those without thrombocytopenia or with thrombocytopenia unrelated to ICIs (6).

Bi-cytopenia was reported in 10.3% of ICI-related cytopenia (3). Nevertheless, cases of ICI-induced pancytopenia are extremely rare in the literature, and only scattered case reports are available (7–13).

Here, we report a case of pancytopenia induced by the PD-1 antibody tislelizumab, which was refractory to G-CSF, thrombopoietin, and thrombopoietin receptor agonists but responded well to steroids and was followed by a cerebral infarction after the resolution of cytopenia.

## Case presentation

**Figure 1:**
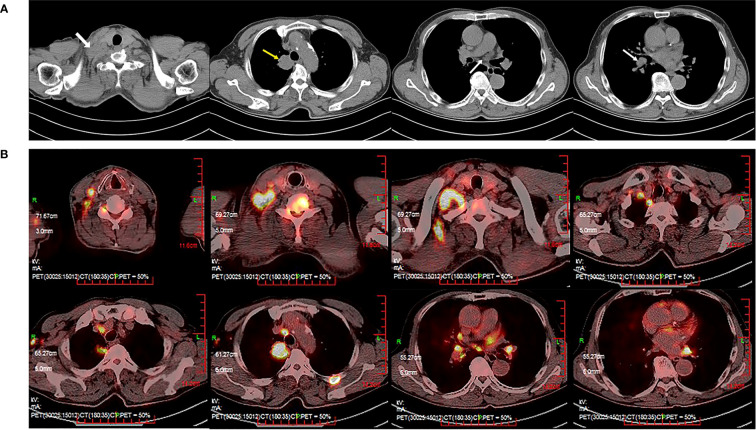
Computed tomography (CT) of the thorax with contrast and positron emission tomography/computed tomography (PET/CT) at the time of diagnosis. **(A)** Thoracic CT revealed multiple masses (the superior lobe of the right lung, supraclavicular region, right hilar and mediastinal lymph nodes). **(B)** PET/CT revealed suspicious primary lung cancer and metastasis.

A 66-year-old male was diagnosed in 2022 with stage IVB (cT2aN3M1c) non-squamous non-small-cell lung cancer (NSCLC). He presented with a supraclavicular mass and pain in the right upper limb on his first visit. Computed tomography (CT) of the chest showed multiple masses in the superior lobe of the right lung, supraclavicular region, and right hilar and mediastinal lymph nodes (**Figure 1A**). Subsequent sodium fluoride (^18^F-NaF) positron emission tomography/computed tomography (PET/CT) confirmed the lesions found on CT and suggested additional metastases involving right axillary lymph nodes, bilateral adrenal glands and multiple bones (cervical vertebras, lumbar vertebras, left iliac crest, left side 4^th^ rib) (**Figure 1B**). Core biopsy of the right supraclavicular mass confirmed the diagnosis of poorly differentiated adenocarcinoma. A panel of 10 genes (*EGFR, KRAS, NRAS, BRAF, PIK3CA, ALK, ROS1, RET, MET, HER2*) tested for driver mutation(s) by amplification refractory mutation system-polymerase chain reaction (ARMS-PCR) was negative for actionable molecular biomarkers. Tested with Dako 22C3 on Dako Autostainer Link 48, the expression level of PD-L1 on tumor cells was 5%.

**Figure 2:**
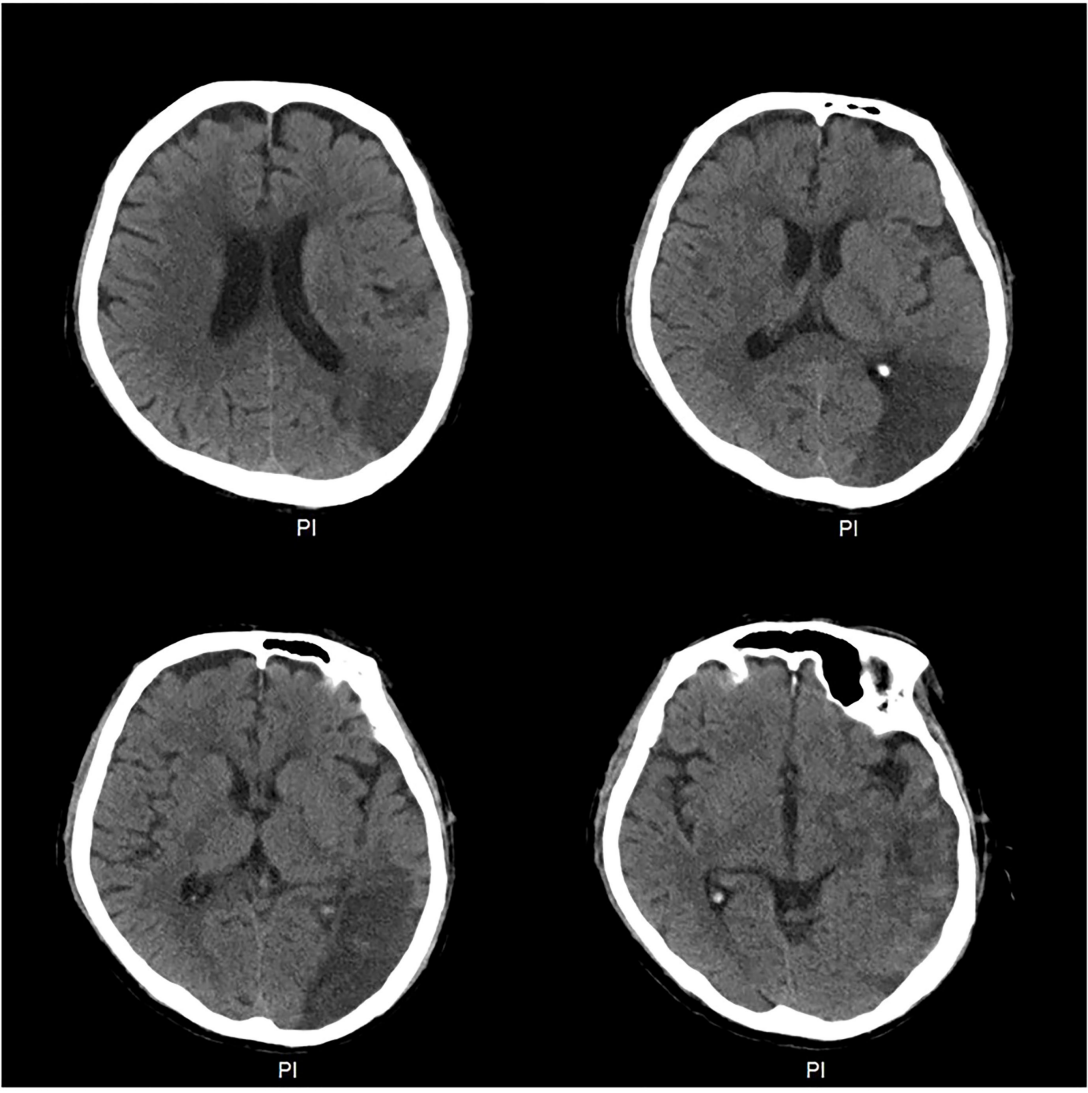
Computed tomography (CT) of the brain at the time of diagnosis of the cerebral infarction. CT of the brain revealed a large area of low density in the left occipital and temporal lobes.

The patient was given one cycle of carboplatin (AUC=4, Day 1) plus pemetrexed (500 mg/m^2^, Day 1) before the gene mutation test. Unfortunately, grade IV rash developed 9 days after chemotherapy. Twenty-one days after the first cycle of chemotherapy, carboplatin was then replaced by cisplatin for the second cycle of therapy, which consisted of cisplatin (75 mg/m^2^, Day 1) and pemetrexed (500 mg/m^2^, Day 1) to avoid possible carboplatin-induced rash. The patient went through the second chemotherapy course fairly well without any serious adverse events. The patient responded to chemotherapy well (partial remission) and reached stable disease during the following two cycles of chemotherapy plus immunotherapy (**Supplemental Figure 1**). Chest CT with intravenous contrast before the third cycle showed the shrinkage of the lung nodules (**Supplemental Figure 1A**). The PD-1 antibody tislelizumab (200 mg, Day 1) was therefore administered to the patient together with pemetrexed and cisplatin (TPC) for the third cycle of therapy. Ten days after the completion of the third cycle regimen, the patient presented with febrile neutropenia with an absolute neutrophil count (ANC) of 120/μL, anemia with a hemoglobin (Hb) level of 75 g/L and thrombocytopenia with a platelet count of 26,000/μL. Two days later, his myelosuppression kept worsening with Hb of 59 g/L and platelet count of 12,000/μL. According to CTCAE5.0, his anemia was graded as III and both neutropenia and thrombocytopenia were grade IV. He was admitted and diagnosed with chemotherapy-induced pancytopenia that resolved after the administration of 9 days of G-CSF and thrombopoietin (TPO) injection, intravenous (IV) antibiotics and platelet and packed red blood cell transfusion. Chest CT with intravenous contrast before the fourth cycle (4^th^C) showed slight further shrinkage of the lung nodules (stable disease) (**Supplemental Figure 1B**). In view of the efficacy and myelosuppression that was supposed to be related to chemotherapeutic agents from TPC regimen, chemotherapy dose reduction was carried out for the 4^th^C therapy that consisted of cisplatin (33 mg/m^2^, Day 1), pemetrexed (280 mg/m^2^, Day 1) and tislelizumab (200 mg, Day 1) administered 30 days after the third cycle. Nine days after the 4^th^C, the patient developed febrile neutropenia (ANC 500/μL) and anemia (Hb 74 g/L). The platelet count was 102,000/μL. Despite timely administration of G-CSF and IV antibiotics for 2 days, his ANC, Hb and platelet count continued to decrease to 410/μL, 66 g/L and 49,000/μL (11 days after the 4^th^C), respectively. He continued G-CSF and IV antibiotics, started TPO and hetrombopag, and received two transfusions of packed red blood cells (4.5 U) and platelets (34 U) during the following 9 days. Eighteen days after the 4^th^C, his ANC, Hb and platelet count were 220/μL, 74 g/L and 7,000/μL, respectively, which prompted us to suspect Hema-irAEs. After a multidisciplinary treatment (MDT) panel discussion, he continued G-CSF, TPO, hetrombopag, and IV antibiotics and started IV steroids with methylprednisolone (1.2 mg/kg) at 21 days after the 4^th^C. Two and 4 more transfusions of packed red blood cells (3 U) and platelets (51 U), respectively, were administered thereafter. His ANC and platelet count reached nadirs of 150/μL (20 days after the 4^th^C) and 3,000/μL (29 days after the 4^th^C), respectively. According to CTCAE5.0, his anemia was graded as III, but both neutropenia and thrombocytopenia were grade IV and life-threatening. His ANC and platelet count increased to 660/μL (3 days after the initiation of methylprednisolone) and 31,000/μL (9 days after the initiation of methylprednisolone), respectively. A peripheral blood smear was then performed 5 days after the initiation of methylprednisolone for abnormalities and showed a decrease of nucleated cells that consisted of predominantly neutrophils (81%), however, negative for malignant cells and parasites (**Supplemental Figures 2A, B**). Methylprednisolone was then tapered slowly from the seventh day of initiation over 6 weeks. His neutropenia and thrombocytopenia resolved on Days 8 (3,870/μL) and 20 (184,000/μL) of methylprednisolone treatment, respectively. His anemia also improved gradually, and he was discharged 40 days after the 4^th^C (Day 20 of methylprednisolone initiation) but returned to the emergency room with symptomatic epilepsy at night on the same day of discharge. Emergency CT of the brain revealed a large area of low density in the left occipital and temporal lobes suggestive of a cerebral infarction (**Figure 2**). Magnetic resonance angiography suggested no significant cerebrovascular stenosis or dilation. Chest CT suggested stable disease (**Supplemental Figure 1C**). He was hospitalized for 8 days and gradually recovered after discharge in terms of the cerebral infarction, leaving his lung cancer unaddressed.

Eighty-five days after the 4^th^C, bone marrow aspiration was pursued to investigate the possible invasion of bone marrow by cancer cells or parasites, which revealed a nonmalignant and parasite-free aspirate marrow specimen (**Supplemental Figure 2**).

**Figure 3:**
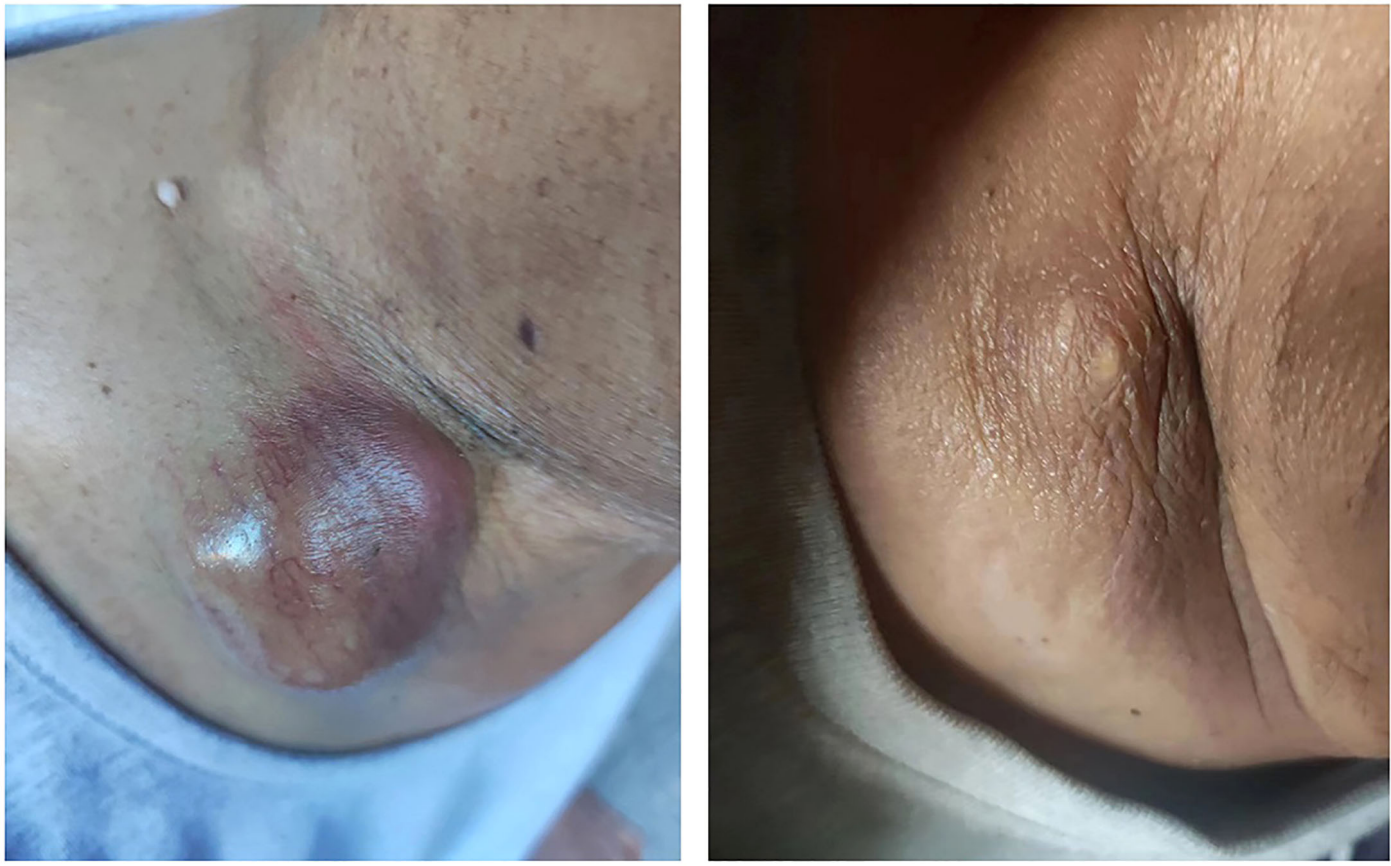
Supraclavicular mass before and after savolitinib treatment. A bulging mass was shown on the right supraclavicular area before treatment with savolitinib (left). The supraclavicular mass shrank dramatically after two weeks of treatment with savolitinib (400 mg/day) (right).

Considering the progression of the lesions (**Supplemental Figure 3A**), he finally agreed to have a core rebiopsy of the right supraclavicular mass 4 months after the 4^th^C, and next-generation sequencing (NGS) was performed to screen for gene mutations, which revealed a mesenchymal epithelial transition (MET) exon 14 skipping mutation (METΔex14). He started savolitinib 400 mg once daily, and significant shrinkage of the right supraclavicular mass was observed 2 days after the initiation of savolitinib. Two weeks after the initiation of savolitinib, the right supraclavicular lesion became flat and invisible from the outside (**Figure 3**). Chest CT showed remarkable remission of all the lesions, and this remission continued, as revealed by chest CT (**Supplemental Figure 3B**) with intravenous contrast 66 days after the initiation of savolitinib. At the time of the preparation of this manuscript, he was on savolitinib and had returned to normal life activities.

## Patient perspective

Our patient reported the following about his experience: I did not expect to recover from the serious episodes of pancytopenia. I felt it important to strictly follow the physician’s suggestions. After taking savolitinib, I soon recovered from the episodes. I appreciated what my physicians had done during the course of my diagnosis and treatment.

## Discussion

Myelosuppression is a common and rare AE of chemotherapy and ICIs, respectively (14). It was reported that the incidence of ICI-induced cytopenia is less than 0.5% in patients administered ICIs (15). Pancytopenia has rarely been reported in patients treated with ICIs (7–13). However, it is very difficult to distinguish chemotherapy-induced myelosuppression from Hema-irAEs when ICIs are administered concurrently with chemotherapy.

**Figure 4:**
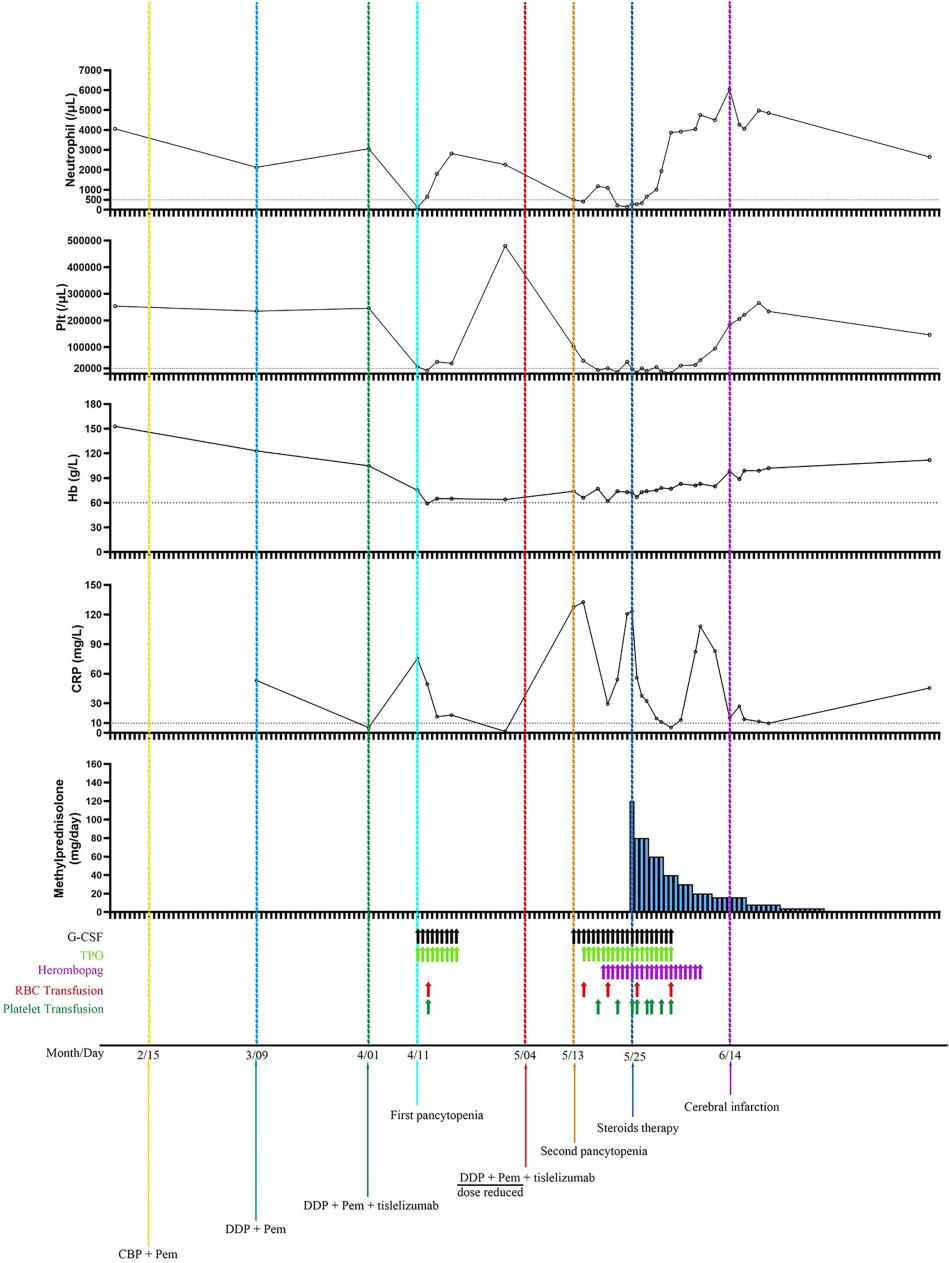
Neutrophil and platelet counts, hemoglobin and CRP concentrations as a function of time and treatments. The first pancytopenia was diagnosed on 10 days after the administration of 3^rd^C (chemotherapy plus tislelizumab) and resolved after the administration of 9 days of G-CSF and TPO injection, and one platelet and packed red blood cell transfusion. The second pancytopenia was diagnosed on 9 days after the 4^th^C (dose reduced chemotherapy plus full dose tislelizumab) and showed resistant to G-CSF, TPO, and transfusions of platelet and packed red blood cells, but responded well to steroids. Plt, platelet; Hb, hemoglobin; CRP, C reactive protein; G-CSF, granulocyte colony-stimulating factor; TPO, thrombopoietin; RBC, red blood cell; CBP, carboplatin; Pem, pemetrexed; DDP, cisplatin.

Taking a comprehensive review of the case, we thought the following supports the diagnosis of Hema-irAEs with tislelizumab but not chemotherapy or other causes induced myelosuppression. (i) The occurrence of his pancytopenia was closely associated with the administration of chemotherapy + tislelizumab, but not with chemotherapy alone (**Figure 4**). (ii) The first two cycles consisting of standard dose platinum-doublet did not induce pancytopenia, while the third cycle that contains standard dose platinum-doublet and tislelizumab triggered serious pancytopenia, and the fourth cycle, despite of sharp dose reduction of platinum-doublet (around 50% of previous dose) + full dose tislelizumab, induced even worse and life-threatening pancytopenia. (iii) The pancytopenia was refractory to G-CSF and thrombopoietin/hetrombopag but responded to 1.2 mg/kg methylprednisolone very well. (iv) Blood smear and bone marrow aspiration did not suggest malignant or parasitic blood or bone marrow environment. (v) The patient did not have any history of myelosuppression or immune disorders.

The cause of ICI-associated cytopenia possibly involves the cytotoxicity of ICI-activated T-cell- and/or B-cell-produced autoantibodies (16). Although there are some publications briefly describing Hema-irAEs, the criteria for the diagnosis and management of Hema-irAEs are not well-established (17–22). In this case, the patient does not have the history of autoimmune diseases or hematological disorders and he received two cycles of chemotherapy after the diagnosis of NSCLC and complained of oral ulcers after chemotherapy without outpatient visits or blood tests. Therefore, it was unclear whether the first two cycles of chemotherapy caused cytopenia. Ten days after the third cycle regimen containing the PD-1 antibody tislelizumab and the previous chemotherapy regimen, the patient developed grade III anemia and grade IV neutropenia and thrombocytopenia that necessitated the transfusion of red blood cells and platelets and injection of G-CSF and TPO. The pancytopenia resolved 9 days later without immunosuppression and remained in remission thereafter, which made it extremely difficult to determine the cause of the pancytopenia. Despite the 12-day delay and significant chemotherapy dose reduction in the fourth cycle regimen, the patient experienced a more severe and longer course of pancytopenia that was resistant to the above management for the first episode of pancytopenia. Despite 11 days of intensive care, the pancytopenia remained unimproved. However, only 3 days after the initiation of methylprednisolone, the patient’s ANC and Hb levels reversed from myelosuppression to 660/μL and 73 g/L, respectively, and his platelet count recovered to 31,000/μL after another 6 days of treatment with methylprednisolone. Finally, his neutropenia and thrombocytopenia resolved on Days 8 and 20 of methylprednisolone treatment, respectively. Therefore, despite the quick resolution of the first pancytopenia episode without immunosuppression, the second pancytopenia episode was extremely resistant to management without steroids but responded very well to subsequent methylprednisolone, strongly suggesting a likely Hema-irAE with tislelizumab. This case implied that higher grade and more refractory Hema-irAEs can occur in patients who resume ICIs after the resolution of previous Hema-irAEs, underlining the importance of the early recognition of Hema-irAEs. The median time to occurrence of ICI-related cytopenia ranges from 6 to 10.1 weeks (3, 15, 17, 20, 23). The patient whose case is described here developed pancytopenia only 10 days after the first dose of tislelizumab. A similar pattern of Hema-irAEs was also reported recently (24). The patient rejected a bone marrow test upon the resolution of neutropenia and thrombocytopenia at 40 days after the 4^th^C. Eighty-five days after the 4^th^C, bone marrow aspiration was performed to investigate the possible invasion of the bone marrow by cancer cells or parasites, and the findings further supported that the patient’s episodes of pancytopenia were Hema-irAEs.

Based on this case and a literature review, to make a timely and accurate diagnosis of ICI-related cytopenia, an MDT panel discussion is strongly suggested, and the following should be covered: the association of cytopenia with ICI administration, the effect of ICI withholding and/or immunosuppression on cytopenia reversal, other reasons that could be causes of the cytopenia, and the presence of autoantibodies reported to be associated with cytopenia.

The patient stopped TPO and hetrombopag when his platelet count reached 50,000/μL. His platelet count never went beyond the upper limit of normal range. However, he suffered a cerebral infarction when his platelet count was within the normal range (184,000/μL). Paradoxically, thrombocytopenia is accompanied by ischemic events (25–29), which indicates risk evaluation for cerebral vascular accident is warranted in cancer patients who experience thrombocytopenia.

Although the specimen for the first molecular testing by PCR was obtained by core biopsy, no known actionable mutation was found, and the METΔex14 revealed by a second testing with NGS strongly argued the importance of dynamic and repeated biopsy and molecular testing, especially by NGS.

## Conclusions

Hema-irAEs are rare with ICIs but can be life-threatening. Early recognition of Hema-irAEs is vital to reduce the potential risks in these patients. An MDT panel discussion can help in the diagnosis and management of Hema-irAEs. Dynamic and repeated biopsy and molecular testing, especially with NGS, should be considered for patients with recurrent and metastatic NSCLC.

## Data availability statement

The raw data supporting the conclusions of this article will be made available by the authors, without undue reservation.

## Ethics statement

The studies involving human participants were reviewed and approved by The Ethical Committee of Zhejiang Provincial People’s Hospital. The patients/participants provided their written informed consent to participate in this study. Written informed consent was obtained from the individual(s) for the publication of any potentially identifiable images or data included in this article.

## Author contributions

H-YG and J-WZ took care of the patient during his hospitalization and prepared the data for this manuscript. Y-SW, Z-NM, X-MZ, and F-WW were members of the diagnosis and treatment group and were involved in the care of the patient. A-HZ and G-QW designed the outline of this case report and guided the preparation of this manuscript. G-QW also led the MDT panel discussion of this case. All authors are aware of and approve this submission. All authors contributed to the article.

## Supplementary material

Supplementary Figure 1 Computed tomography (CT) of the thorax during the course of anticancer therapy and management of pancytopenia **(A)** Two cycles of chemotherapy led to partial remission of lung cancer. **(B)** One cycle of dose-reduced chemotherapy+tislelizumab led to further shrinkage of multiple nodules. **(C)** Forty days after the second dose of tislelizumab, CT of the thorax indicated stable disease.

Supplementary Figure 2 Blood smear and bone marrow examination **(A,B)** Blood smear and **(C, D)** bone marrow aspiration revealed non-malignant and parasite-free environment.

Supplementary Figure 3 Computed tomography (CT) of the thorax before and after the administration of savolitinib **(A)** Four months after the second dose of tislelizumab, computed tomography (CT) of the thorax indicated progressive disease. **(B)** Chest CT showed partial remission of the lesions after two weeks of savolitinib treatment.
