# Supplementary figures and images for: Case Report: Life-threatening pancytopenia with tislelizumab followed by cerebral infarction in a patient with lung adenocarcinoma
Source: Front Immunol. 2023 Jul 25;14:1148425. doi: 10.3389/fimmu.2023.1148425 (PMC10409480; doi:10.3389/fimmu.2023.1148425)

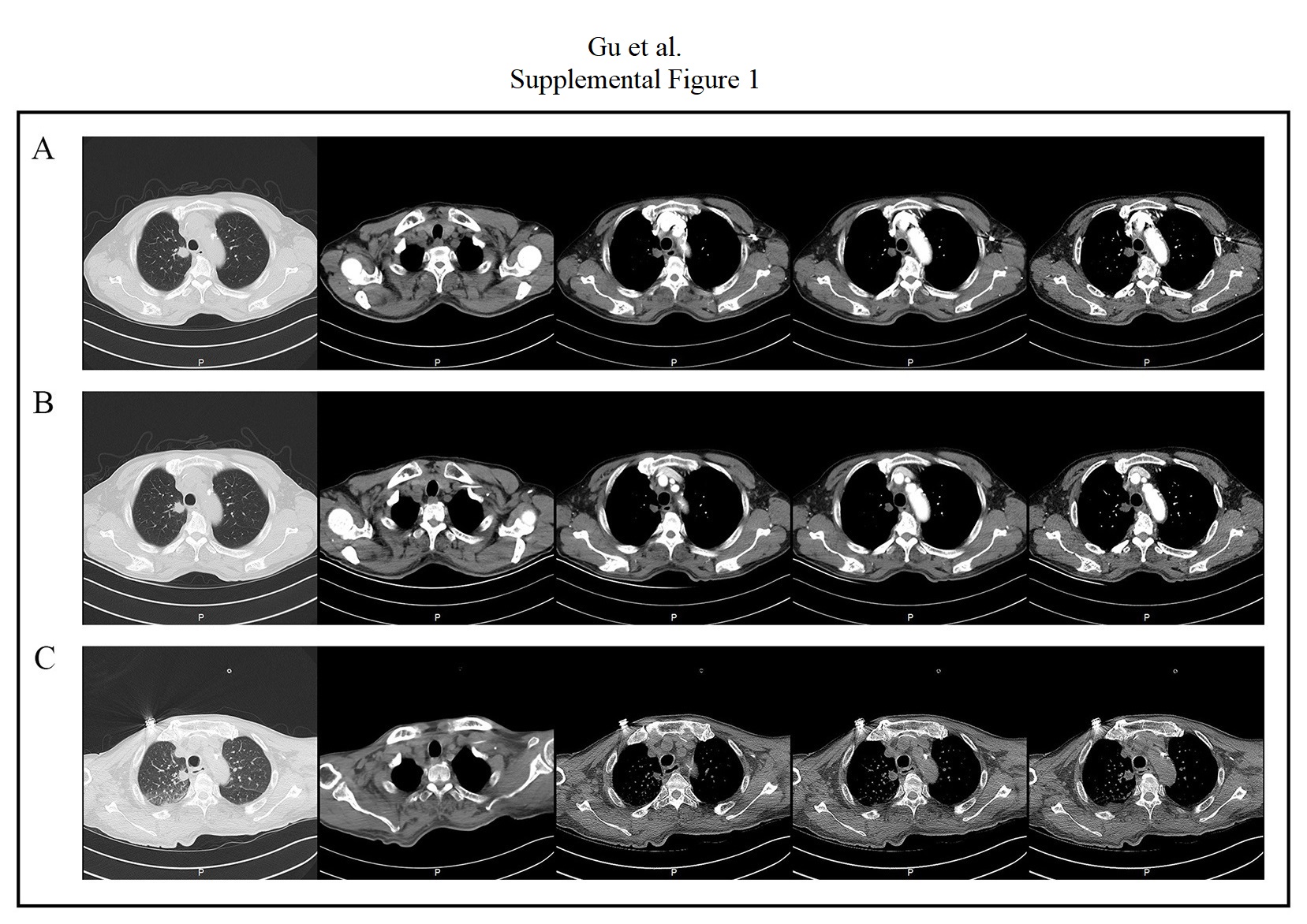

Supplement: Supplementary Figure 1 — Computed tomography (CT) of the thorax during the course of anticancer therapy and management of pancytopenia (A) Two cycles of chemotherapy led to partial remission of lung cancer. (B) One cycle of dose-reduced chemotherapy+tislelizumab led to further shrinkage of multiple nodules. (C) Forty days after the second dose of tislelizumab, CT of the thorax indicated stable disease. [file Image_1.jpeg]

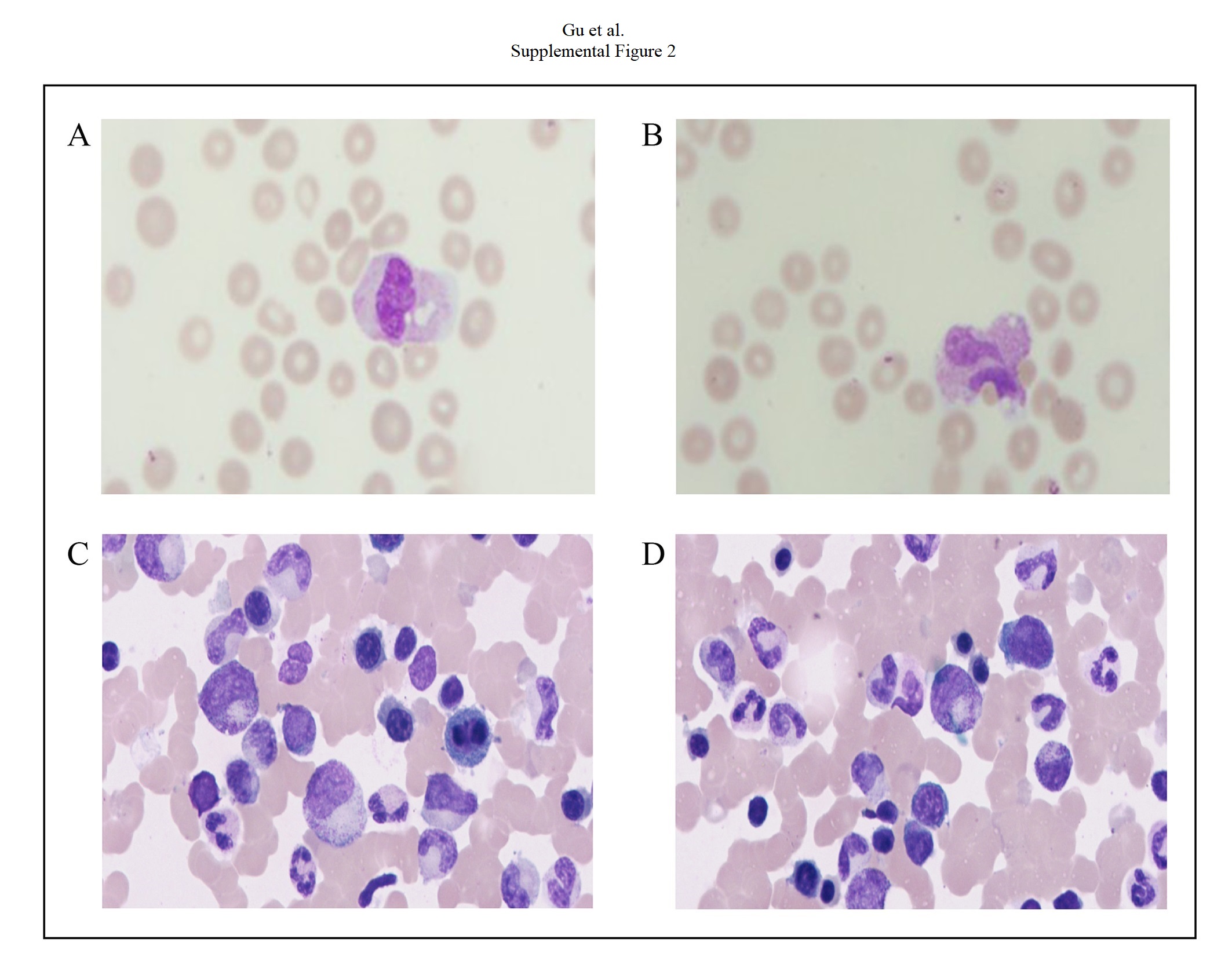

Supplement: Supplementary Figure 2 — Blood smear and bone marrow examination (A,B) Blood smear and (C, D) bone marrow aspiration revealed non-malignant and parasite-free environment. [file Image_2.jpeg]

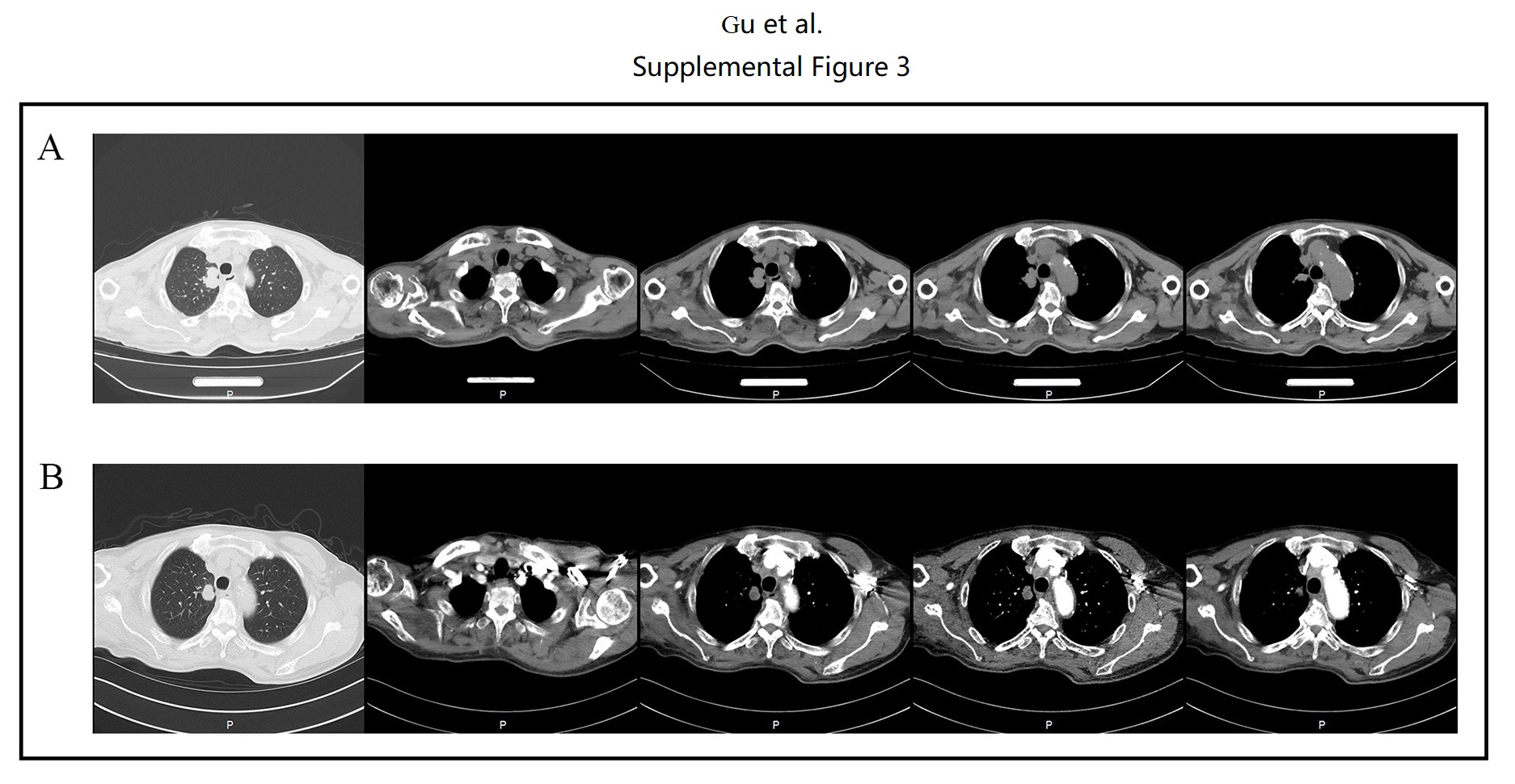

Supplement: Supplementary Figure 3 — Computed tomography (CT) of the thorax before and after the administration of savolitinib (A) Four months after the second dose of tislelizumab, computed tomography (CT) of the thorax indicated progressive disease. (B) Chest CT showed partial remission of the lesions after two weeks of savolitinib treatment. [file Image_3.jpeg]

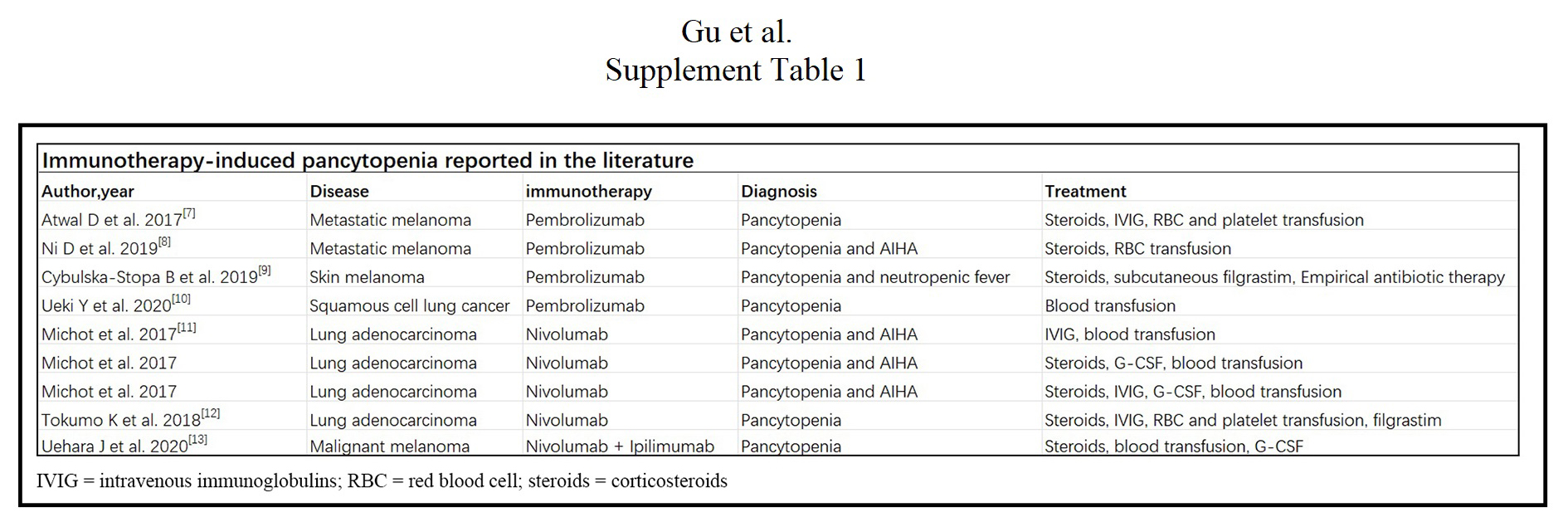

Supplement: Supplementary file 4 [file Image_4.jpeg]
